# A Dynamic QoS Mapping Algorithm for 5G-TSN Converged Networks Based on Weighted Fuzzy C-Means and Three-Way Decision Theory

**DOI:** 10.3390/s25216648

**Published:** 2025-10-30

**Authors:** Yuhang Wu, Fangmin Xu, Lina Ning, Xiaokai Liu, Hongyuan Chen, Xingbo Lu, Chenglin Zhao

**Affiliations:** 1 School of Information and Communication Engineering, Beijing University of Posts and Telecommunications, Beijing 100876, China; yuhang_wu@bupt.edu.cn (Y.W.); xufm@bupt.edu.cn (F.X.); clzhao@bupt.edu.cn (C.Z.); 2 The Intelligent Gaming and Decision-Making Laboratory, Beijing 100091, China; xingbo_lu@sina.com; 3 School of Mechanical and Electrical Engineering, Beijing Information Science and Technology University, Beijing 102206, China; 4 School of Electronic Engineering, Beijing University of Posts and Telecommunications, Beijing 100876, China; hongyuan_chen@bupt.edu.cn

**Keywords:** time-sensitive networking (TSN), 5G, quality of service (QoS) mapping, flow clustering, three-way decisions, load balancing

## Abstract

To ensure end-to-end Quality of Service (QoS) management in 5G-TSN converged networks, this paper proposes a dynamic weighted QoS mapping method based on Weighted Fuzzy C-Means and Three-Way Decisions (WFCM-TDwQM). The WFCM algorithm is employed to cluster Time-Sensitive Networking (TSN) flows based on their QoS attributes, reducing computational complexity. A three-way decision-based method is used to assign a reasonable and approximate set of 5G QoS Identifier (5QI) values to each cluster. Finally, dynamic weights are adjusted by considering QoS similarity and the residual load rate, enabling the system to adapt to network load changes. The experimental results show that, compared with three other mapping algorithm combinations, WFCM-TDwQM not only ensures end-to-end QoS consistency but also achieves better load balancing under varying network loads. Moreover, its mapping performance is evaluated under different network scenarios.

## 1. Introduction

Although wired Ethernet has been widely adopted in modern industrial communication systems due to its high reliability and mature technological foundation, it suffers from limited flexibility and high installation and maintenance costs [1]. These limitations have created opportunities for the development of wireless communication systems such as Wi-Fi, 4G, and, more recently, 5G [2,3,4]. In wireless communication systems, various devices must efficiently interconnect within the constraints of limited radio spectrum resources [5]. With the continuous evolution of wireless and cellular communication technologies, the capacity and coverage of industrial communication systems have improved significantly. By integrating Time-Sensitive Networking (TSN) from wired networks with 5G technology from wireless domains, communication systems can achieve enhanced flexibility while meeting the stringent requirements of industrial applications for high bandwidth and low latency [6,7,8,9]. In the architecture of 5G-TSN converged networks, the 5G system (5GS) acts as a transparent logical bridge, supporting the necessary interfaces and protocols required to participate in TSN networks, enabling efficient and unified industrial communication [10,11].

In integrated wireless and wireless networks, each (sub)network may operate using different protocols, and the underlying data flows may exhibit different characteristics of Quality of Service (QoS) [12,13,14,15]. To achieve seamless and unified communication across such heterogeneous networks, it is necessary to systematically map the QoS properties of data flows between different (sub)networks [16]. One of the key challenges in such environments is the estimation of end-to-end QoS for data flows [17]. A commonly adopted approach to evaluating the overall performance of heterogeneous networks involves quantifying the impact of each participating application and access technology [18,19,20]. Unfortunately, the 3GPP specification does not define a standard mapping between the QoS attributes of 5G and TSN networks [21]. Designing such a mapping is a complex task, yet it is crucial, as it can significantly affect the end-to-end service quality of traffic flows in heterogeneous TSN–5G integrated networks [22,23].

Currently, mainstream QoS mapping algorithms can generally be categorized into three types: table-based mapping, function-based mapping, and clustering-based mapping.

Table-based QoS mapping uses predefined static mapping tables that specify relationships between different QoS domains [24]. For example, Satka et al. [25] proposed a method that maps the default priority values of 5G frames to the priority code point (PCP) values of TSN frames and vice versa, enabling interconversion between 5G and TSN frames. However, this type of mapping is inherently static and lacks adaptability to changing network conditions; under heavy network loads, it can easily lead to congestion in specific QoS queues due to its static nature [26].

Function-based QoS mapping establishes mathematical relationships between QoS parameters in heterogeneous networks to perform mapping. Shaikhli et al. [12] proposed a framework that supports end-to-end QoS in heterogeneous networks, classifying incoming traffic into two distinct QoS categories based on application type and QoS requirements. In [27], a QoS-aware algorithm was developed to systematically map TSN traffic to 5G domains based on application constraints such as deadlines, jitter, and bandwidth. However, function-based mapping remains largely static and lacks flexibility in adapting to dynamic network conditions.

Cluster-based QoS mapping first aggregates traffic flows with similar QoS characteristics into cluster flows, then performs unified QoS mapping for each cluster [28,29]. Kumar D et al. [30] designed a dynamic flow clustering scheme based on a rough K-means algorithm that adapts clustering based on network environments. However, this method is limited to fixed QoS class mappings. Cai Y et al. [31] proposed IKM-RQM, a solution for low-latency transmission and resource management in TSN–5G systems. This approach relies on a clustering-based TSN QoS mapping table and a 5G QoS Identifier (5QI) mapping table generated using a distance-based rough set method. However, the mapping success rate of IKM-RQM decreases under high network load, and the distance-based RQM approach exhibits limited flexibility, as the setting of threshold pairs relies heavily on subjective judgment.

References [32,33,34,35] conducted a series of studies on the network architecture, key technologies, research challenges, and application scenarios of 5G–TSN converged networks. Reference [36] proposed an effective method for mapping 5G QoS flows to the priority queues of time- and wavelength-division multiplexed passive optical networks (TWDM-PONs). Considering the latency tolerance of services and the load conditions of the network, this study mapped 22 standardized 5G QoS Identifiers (5QIs) to the (high, medium, and low) priority queues of the PON. Furthermore, Zhang et al. [37] proposed a QoS-aware dynamic scheme to enable interconnection between 5G and TSN networks, with a primary focus on virtual network function (VNF) mapping. They formulated a mixed-integer linear programming (MILP) model incorporating time-sensitive constraints and developed a heuristic algorithm for VNF mapping and scheduling within the 5G–TSN integrated environment.

To alleviate the contradiction between QoS consistency and load balancing in the QoS mapping of 5G-TSN converged networks, this study proposes a dynamic weighted QoS mapping method that combines Weighted Fuzzy C-Means clustering with a three-way decision-making mechanism (WFCM-TDwQM). The proposed approach first applies WFCM to cluster TSN flows based on their QoS attributes, thereby reducing the computational complexity of the subsequent mapping process. A three-way decision-based region partitioning strategy is then used to assign the available 5QIs to core and boundary domains for each cluster, providing a constrained and reasonable candidate set for mapping. Based on the clustering results and the partitioned 5QI regions, a dynamic weight-driven QoS mapping procedure is finally performed, which ensures QoS consistency while improving the balance of traffic load across the network. The primary contributions of this work are summarized as follows:

1. This paper employs WFCM clustering to capture variations in different QoS attributes across traffic flows effectively. During the iterative clustering process, attribute weights are dynamically adjusted based on their contribution to intra-cluster differentiation. Attributes with greater impact are assigned higher weights in the distance calculation. This adaptive weighting mechanism enhances clustering performance and reduces the computational complexity of the subsequent QoS mapping stage.
2. A three-way decision-based evaluation mechanism is introduced to partition the 5QI set into core and boundary domains for each flow cluster. This method takes into account the relative membership degrees of 5QIs to each cluster and calculates threshold pairs along with associated evaluation metrics. By avoiding reliance on manually set absolute thresholds, the partitioning process becomes more adaptive and robust. This stage effectively prepares the foundation for balanced and flexible mapping while ensuring QoS consistency.
3. Based on the results of WFCM clustering and three-way partitioning, WFCM-TDwQM performs QoS mapping from TSN flows to 5QI parameter groups using a dynamic weighting strategy. The mapping weight considers both the residual capacity of each 5QI and its QoS similarity to the target flow. This approach achieves a better balance between load distribution and QoS alignment, particularly under varying network load conditions.

The proposed WFCM-TDwQM approach effectively addresses the end-to-end QoS mapping challenge in 5G-TSN converged networks by combining weighted fuzzy clustering with dynamic decision-based weight adjustment. These contributions advance the intelligent management of heterogeneous network resources and enhance service continuity across converged industrial scenarios. The remainder of this article is organized as follows: Section 2 introduces the 5G-TSN converged network architecture and the QoS model, laying the foundation for the proposed method. Section 3 presents the design of the WFCM-TDwQM algorithm, including traffic clustering based on composite QoS metrics and dynamic 5QI allocation using a three-way decision mechanism. Section 4 details the experimental setup, performance metrics, and comparative results under various network load conditions. Finally, Section 5 concludes this paper and discusses potential directions for future research.

## 2. 5G–TSN Converged Network

### 2.1. 5G–TSN Network Model

In the 5G–TSN converged network architecture, the 5GS operates as a transparent bridge device that supports TSN functionality. The 5G standard leverages its native QoS framework to manage traffic flows, which can be differentiated using corresponding 5QI values [38]. To ensure interoperability between TSN and 5G domains, the logical bridge in 5G includes TSN Translator (TT) functions that operate across both the user plane and the control plane [39]. In the user plane, the TSN Translator is composed of a network-side TT (NW-TT) and a device-side TT (DS-TT), which provide ingress and egress adaptation support. In the control plane, the 5G architecture achieves coordinated configuration, QoS mapping, and management interoperability through the interaction between the TSN Application Function (AF) and the Centralized Network Configuration (CNC) entity [40].

**Figure 1:**
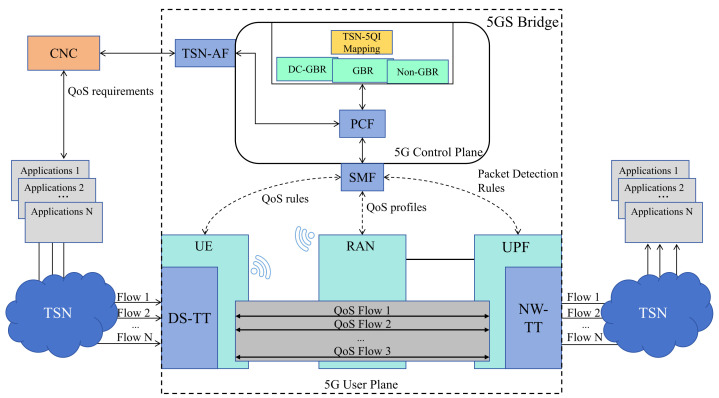
Diagram of QoS mapping in 5G-TSN converged networks.

As shown in Figure 1, the 5GS obtains the QoS requirements of the TSN flows from the CNC through the TSN-AF in the control plane. Based on this information and the pre-configured 5QI profiles, the QoS mapping algorithm is used to create a QoS mapping table between TSN and the 5GS. Within the algorithmic procedure, flows with similar QoS characteristics are merged into a single aggregated flow [41]. Since the number of aggregated flows is significantly smaller than the number of original flows, the computational complexity of the subsequent QoS mapping process is greatly reduced [42]. Based on this mapping table, a Policy Control Function (PCF) selects an appropriate QoS profile for each flow. The PCF then instructs the Session Management Function (SMF) to establish the corresponding 5G QoS flows, thereby completing the QoS mapping between TSN and the 5GS [43].

On the UE side, TSN flows are allocated to the corresponding 5G QoS flows by DS-TT according to the issued QoS rules. These QoS flows are subsequently scheduled in the RAN according to their assigned QoS profiles. Resource allocation is carried out within the Radio Access Network (RAN), where the MAC-layer scheduler in the 5G system assigns radio resources according to the defined QoS profiles. On the UPF side, NW-TT restores TSN flows from QoS flows and handles them based on Packet Detection Rules. Using the allocated resources, traffic is transmitted through the 5GS with deterministic delivery guarantees, thus enabling end-to-end QoS support [44,45].

### 2.2. QoS Model

At the user plane, a total of N TSN traffic flows of various types are injected from the TSN network into the 5G bridge. Each flow is represented as a multivariate vector of QoS requirements: $f_{i}=\left[f_{i,1},f_{i,2},\ldots ,f_{i,D}\right]$, where 1≤n≤N, D denotes the number of QoS parameters, and $f_{i,d}$ represents specific QoS features such as priority, transmission guarantee, latency, data volume, and packet loss rate. These QoS parameters are assumed to be known to the 5G system, typically obtained via the TSN AF. Accordingly, the set of traffic flows within a given time slot can be denoted as follows: $F=\left\{f_{1},f_{2},\ldots ,f_{N}\right\}$. Before being processed by the 5GS for QoS mapping, the TSN flows are pre-clustered into C aggregated flow groups, where C<N. Each group contains flows with similar QoS characteristics. The $af_{k}$ aggregated flow is denoted as follows: $\phi_{k}=\left\{f_{k1},f_{k2},\ldots ,f_{nk}\right\}$, where nk is the number of original flows contained in the aggregated flow $af_{k}$. The complete set of aggregated flows can thus be expressed as follows: $AF=\left\{\phi_{1},\phi_{2},\ldots ,\phi_{C}\right\}$.

In the 5GS, a QoS flow represents the finest granularity of service differentiation within a Protocol Data Unit (PDU) session. Each QoS flow between the User Equipment (UE) and the User Plane Function (UPF) is characterized by a set of QoS attributes, including resource type, priority level, packet delay budget (PDB), packet error rate (PER), and maximum data burst volume (MDBV) [46]. These attributes are encapsulated in QoS profiles and referenced using the 5G QoS Identifier (5QI). Therefore, QoS mapping between TSN and 5G involves assigning a corresponding 5QI to each TSN traffic flow. The 3rd Generation Partnership Project (3GPP) specification defines a set of standardized 5QI values, which are commonly used for typical services in mobile networks. This set can be extended with non-standardized 5QI values, allowing network operators to customize QoS characteristics to meet specific service requirements. Each supported 5QI in the system can be represented as a vector of QoS attributes, expressed as follows: $I_{j}=\left[I_{j,1},I_{j,2},\ldots ,I_{j,D}\right]$, for 1≤j≤L, where $I_{j,d}$ denotes the d-th QoS attribute index for the j-th 5QI. These indices correspond to the same QoS parameters as defined for TSN traffic flows, such as priority, resource type, PDB, MDBV, and PER. It is worth noting that not all 5QI types include a complete set of parameters. Specifically, 5QIs with a Guaranteed Bit Rate (GBR) resource type do not define the MDBV parameter, indicated by $I_{j,4}=-1$. Meanwhile, non-GBR 5QIs typically lack both PDB and MDBV settings, denoted as $I_{j,3}=-1$ and $I_{j,4}=-1$, respectively.

To enable flexible and accurate QoS mapping in 5G–TSN converged networks, the goal of clustering-based dynamic QoS mapping is to reduce, as much as possible, the discrepancy between 5G QoS classes and TSN traffic classes. That is, the assigned QoS levels after mapping should closely approximate the original QoS levels, thereby minimizing potential degradation in transmission quality due to mismatched service classes. Meanwhile, when the network experiences heavy traffic load, the dynamic mapping scheme should be capable of adjusting the assignment of traffic flows to 5QI queues in a timely manner. This helps prevent queue buildup caused by static mappings and reduces additional network latency under congested conditions.

## 3. Algorithm Design

**Figure 2:**
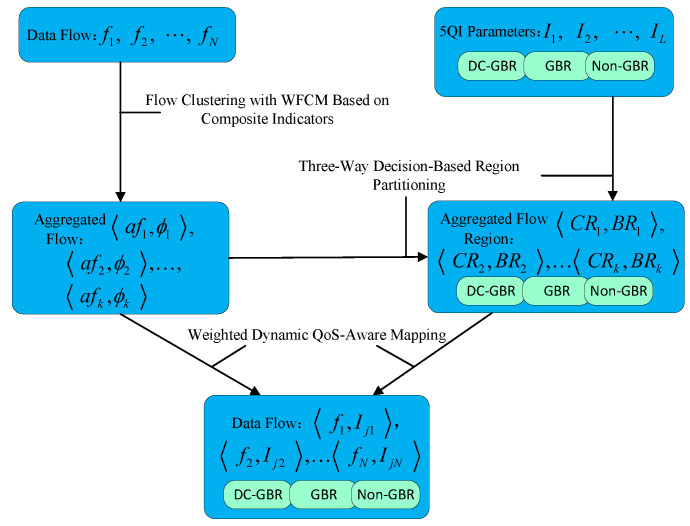
Overall algorithm flow.

In a 5G–TSN converged network, the input consists of N TSN traffic flows, each described by D QoS attributes. The 5G system is pre-configured with L standardized 5QI entries, each also characterized by D QoS parameters. After flow clustering, the N TSN flows are grouped into C aggregated flow clusters. Each cluster is associated with a cluster center $af_{k}$ and a corresponding set of flows $\phi_{k}$, where k=1,…,C. Based on the cluster center $af_{k}$ of each aggregated flow, the set of L 5QIs is partitioned using a three-way decision mechanism. For each aggregated flow cluster, this results in two subsets of 5QIs: a core region set $CR_{k}$ and a boundary region set $BR_{k}$, where k=1,…,K. Finally, each flow in the aggregated set $\phi_{k}$ is mapped to a 5QI in the corresponding core region set $CR_{k}$, provided that the selected 5QI has sufficient residual capacity. If all 5QIs in $CR_{k}$ are fully utilized, the flow is then mapped to a 5QI in the boundary region set $BR_{k}$ that still has available capacity. The output of this process is a complete mapping from each TSN flow $f_{i}$ (where i=1,…,N) to a 5QI $I_{ni}$, with $n_{i}\in \left\{1,\ldots ,L\right\}$, thereby accomplishing the QoS mapping of TSN traffic onto 5G QoS classes. The overall workflow of the algorithm is illustrated in Figure 2.

### 3.1. WFCM-Based Traffic Clustering

#### 3.1.1. Computation of Composite QoS Weights in TSN Networks

##### CRITIC–Entropy-Based Weight Calculation

Commonly used methods for computing attribute weights include the entropy weight method and the Criteria Importance Through Intercriteria Correlation (CRITIC) method. The entropy weight method reflects the degree of dispersion among QoS attribute data, while the CRITIC method takes into account both the correlation between QoS attributes and the contrast intensity among values. The integration of these two methodologies produces a more balanced and informative weight distribution [47].

It should be noted that both entropy and standard deviation serve as indicators of data dispersion, which means that they are equally important in characterizing attribute variability. To leverage the strengths of both approaches, this study employs an additive combination strategy. The formula for the combined weight computation that integrates the CRITIC and entropy methods is shown as follows:

(1) $$CE_{j}=\left(\sigma_{j}^{2}+e_{j}\right)\sum_{i=1}^{D}\left(1-q_{ij}\right)$$

(2) $$w_{j}^{a}=\frac{CE_{j}}{\sum_{j=1}^{D}CE_{j}}$$

Here, $\sigma_{j}^{2}$ denotes the standard deviation of the QoS attribute values in the j-th dimension across all traffic flows; $e_{j}$ represents the information entropy of the QoS attribute in the j-th dimension; and $q_{ij}$ is the correlation coefficient between the QoS attributes in the i-th and j-th dimensions.

##### Fisher Criterion-Based Weight Calculation

In binary classification, the traditional Fisher criterion aims to minimize the within-class scatter while maximizing the between-class scatter. In a one-dimensional sample space, the corresponding binary classification Fisher function is shown as follows:

(3) $$F_{2,j}=\frac{\sum_{i=1}^{2}\sum_{k=1,k\neq i}^{2}{\left(\mu_{i}-\mu_{k}\right)}^{2}}{\sum_{i=1}^{2}\sum_{k=1,k\neq 1}^{2}\left(s_{i}^{2}+s_{k}^{2}\right)}=\frac{{\left(\mu_{1}-\mu_{2}\right)}^{2}}{\left(s_{1}^{2}+s_{2}^{2}\right)}$$

Here, $\mu_{k}$ and $s_{k}^{2}$ represent the mean and within-class scatter of the samples belonging to the class group k on attribute j, respectively. The Fisher function evaluates the contribution of attribute j to the binary classification. Extending the above form to the multi-class case, its Fisher function is expressed as follows:

(4) $$F_{C,j}=\frac{\sum_{i=1}^{C}\sum_{k=1,k\neq i}^{C}{\left(\mu_{i}-\mu_{k}\right)}^{2}}{\sum_{i=1}^{C}\sum_{k=1,k\neq 1}^{C}\left(s_{i}^{2}+s_{k}^{2}\right)}$$

By replacing the within-class scatter in the denominator with the within-class sample variance, we obtain Equation (5). This reduces the impact of the number of within-class samples on the Fisher function and makes the form of the denominator more reasonable. Based on the current clustering result, the mean and variance of each attribute are computed for each cluster. To evaluate the discriminative power of each attribute, pairwise comparisons are performed between clusters; the Fisher linear discriminant ratio is calculated for each attribute by comparing the inter-cluster mean differences and the intra-cluster variances [48] :

(5) $$F_{j}=\frac{\sum_{i=1}^{C}\sum_{k=1,k\neq i}^{C}{\left(\mu_{i}-\mu_{k}\right)}^{2}}{\sum_{i=1}^{C}\sum_{k=1,k\neq 1}^{C}\left(\sigma_{i}^{2}+\sigma_{k}^{2}\right)}$$

The attribute weight for each QoS dimension is derived from its Fisher discriminant ratio, as follows:

(6) $$w_{j}^{b}=\frac{F_{j}}{\sum_{j=1}^{D}F_{j}}$$

##### Composite Weight Calculation

CRITIC–entropy weights measure the internal and external mathematical characteristics of sample attribute values; multi-class Fisher weights reflect the Fisher contribution of each attribute to classification. The former is calculated during clustering initialization, while the latter is computed during the iteration process. By combining them through a product accumulation normalization method, as shown in Equation (7), the resulting composite weights both account for the static mathematical features of the samples and dynamically adjust the weights of each attribute based on grouping. This increases the weights of attributes beneficial to the grouping, thus improving the quality of the grouping.

(7) $$w_{j}=\frac{w_{j}^{a}\cdot w_{j}^{b}}{\sum_{j=1}^{D}w_{j}^{a}\cdot w_{j}^{b}}$$

#### 3.1.2. Attribute-Weighted Fuzzy C-Means Clustering for Traffic Flows

The aforementioned comprehensive weighting method is incorporated into the attribute-weighted fuzzy C-means (FCM) clustering algorithm. The overall procedure is summarized as follows. Initially, the QoS indicators of TSN traffic flows are discretized and normalized to ensure consistency across different attributes. An integer C is then selected to represent the number of clusters, satisfying 2≤C≤N−1. In addition, the fuzzy control parameter m (commonly set to 2) and a convergence threshold ε (set to $e^{-2}$ in this work) are determined. The fuzzy membership matrix U is initialized, where each element of the matrix is constrained within the interval $\mu_{ik}\in \left[0,1\right]$. Subsequently, the CRITIC–entropy weights are calculated, and based on Equation (10), the initial cluster centers $c_{k}$ (1≤c≤C) are obtained. For each data element, its cluster assignment is determined according to the maximum membership degree in the matrix U. This step provides an initial partition of traffic flows into candidate clusters, and the iteration begins. In each iteration, based on the class grouping from the previous iteration, the contribution of each attribute component is evaluated using Fisher’s linear discriminant ratio. The Fisher criterion weights are derived accordingly, thereby capturing the relative discriminative power of individual QoS indicators across different clusters [49]. By combining these weights, the comprehensive indicator weight is finally obtained. The distance between each data sample and the cluster centers is then computed using a weighted Euclidean metric, defined as follows:

(8) $$d_{ik}^{w}=\sqrt{\sum_{d=1}^{D}\left(w_{d}\cdot {∥f_{i,d}-af_{k,d}∥}^{2}\right)}$$

where $w_{d}$ denotes the weight of the *d*-th QoS attribute. Based on these distances, the membership function is updated as follows:

(9) $$\mu_{ik}=\frac{1}{\sum_{p=1}^{C}{\left(\frac{d_{ik}^{w}}{d_{ip}^{w}}\right)}^{\frac{2}{m-1}}}\;$$

The cluster centers are then recalculated according to the following:

(10) $$c_{k}=\frac{\sum_{i=1}^{N}\mu_{ik}^{m}\cdot x_{i}}{\sum_{i=1}^{N}\mu_{ik}^{m}}\;$$

At the end of each iteration, the convergence of the algorithm is assessed by comparing the difference between the cluster centers before and after iteration. If the change is smaller than the threshold ε, the clustering process terminates; otherwise, the procedure returns to the step of computing the initial cluster centers $c_{k}$ and repeats the subsequent updates until convergence is achieved.

It is worth noting that this iterative process adaptively adjusts both the membership degrees and the cluster centers, ensuring that the clustering outcome reflects not only the intrinsic similarity of traffic flows but also the varying significance of different QoS attributes. This property is essential for providing a reliable foundation for subsequent QoS mapping in heterogeneous 5G-TSN environments.

### 3.2. Dynamic Weighted QoS Mapping Based on Three-Way Decisions

#### 3.2.1. Three-Way Decision-Based 5QI Region Partitioning

According to the 3GPP 5G specifications, there are three defined resource types: Delay-Critical GBR (DC-GBR), Guaranteed Bit Rate (GBR), and non-GBR. The Policy Control Function (PCF) computes an approximate set of 5QIs for time-sensitive (TS) flows within the DC-GBR resource type. For non-time-sensitive flows, including AVB flows, best-effort (BE) flows, and other general traffic types, the approximate set is computed using 5QIs from the GBR and non-GBR categories [50]. To avoid priority confusion before and after QoS mapping, as shown in Table 1, QoS class mapping is established by correlating the priority code point (PCP) of TSN traffic with the resource type of the available 5QI entries.

Traditional static QoS mapping methods are incapable of dynamically adapting their strategies or expanding mapping tables in response to changes in network load, which may lead to congestion within specific QoS categories. To enhance the flexibility of the mapping process, rough set theory is employed to expand the suboptimal 5QI set, thereby improving the efficiency of resource allocation.

However, determining how to effectively divide the optimal 5QI set (corresponding to the core region) and the suboptimal 5QI set (corresponding to the boundary region) remains a key challenge in achieving flexible load balancing while ensuring QoS consistency. A distance-based partitioning method is employed, which uses a minimum distance threshold *tl* and a relative distance threshold *th* to delineate the core and boundary regions. First, for each 5QI element $I_{j}$, the distance $d_{jk}$ to all clustering centers $af_{k}$ is calculated, along with its minimum value $d_{jm}$. The partitioning procedure proceeds as follows: If $d_{jm}\leq tl$, the 5QI element $I_{j}$ is assigned to the core region $CR_{m}$ corresponding to cluster center $c_{m}$; otherwise, it is assigned to the boundary region $BR_{m}$. For the remaining cluster centers not yet evaluated for $I_{j}$, if $d_{jk}\leq tl$, then $I_{j}$ is assigned to the core region $CR_{k}$; otherwise, the ratio $p_{km}=d_{jk}/d_{jm}$ is calculated. If $p_{km}\leq th$, then $I_{j}$ is assigned to the boundary region $BR_{k}$; otherwise, it is assigned to the negative region $DR_{k}$ [31].

Although the distance-based partitioning method is relatively simple in terms of decision logic, it still relies on the manual configuration of two threshold parameters. To address this limitation, this paper incorporates the three-way decision mechanism used in three-way C-means clustering into the 5QI region partitioning process [51], resulting in a three-way decision-based partitioning method. As with the previous approach, the distances $d_{jk}$ between each 5QI element $I_{j}$ and all clustering centers $af_{k}$ are first computed. Then, the membership degree of each 5QI element with respect to each clustering center is calculated using the following formula:

(11) $$\mu_{jk}^{*}=\frac{1}{\sum_{p=1}^{C}{\left(\frac{d_{jk}}{d_{jp}}\right)}^{\frac{2}{m-1}}}$$

The relative membership degree $g_{jk}$ is defined by computing the ratio between the membership value $\mu_{jk}^{*}$ and the maximum membership value among all clusters for a given 5QI element, as formulated below:

(12) $$g_{jk}=\frac{\mu_{jk}^{*}}{\mu_{jn}^{*}}$$

Here, $\mu_{jn}^{*}$ denotes the maximum value of $\mu_{jk}^{*}$, ensuring that $g_{jk}\leq 1$ is always satisfied. Based on the relative membership degrees of each sample, a threshold $\delta_{a}$ is derived for logical decision-making during numerical filtering. The calculation is given by the following [52]:

(13) $$\delta_{a}=\frac{1}{L_{a}}\sum_{j\in J_{a}}g_{jp}-g_{jq}$$

Here, $a\in \left\{1,2,3\right\}$ corresponds to the resource types DC-GBR, GBR, and non-GBR, respectively. $L_{a}$ denotes the number of 5QI entries with resource type *a*; $J_{a}$ is the corresponding 5QI set; and $g_{jp}$ and $g_{jq}$ denote the highest and second highest relative membership degrees for 5QI element $I_{j}$. For each 5QI $I_{j}$ of type a, all associated relative membership degrees $g_{jk}$ are compared with the threshold $\delta_{a}$, and a binary logical value is defined as $L_{jk}=g_{jk}>\delta_{a}$. By employing these logical values, the regions of this 5QI can be succinctly ascertained. When there is only one $L_{jk}=1$, it signifies that the 5QI is associated with the CR of the aggregated flow k; conversely, when there are multiple different ones $L_{jk}=1$, it denotes that the 5QI pertains to the BR of multiple aggregated flows. This distinction exemplifies the partitioning characteristic inherent in rough set theory, whereby an element belongs either to the CR of a single class or to the BR shared by multiple classes.

Next, based on the relative membership degrees and the binary logical values, both the evaluation function and the pair of partitioning thresholds are derived. The evaluation function is defined as follows:

(14) $$v_{jk}=\frac{g_{jk}}{\sum_{k=1}^{C}L_{jk}}$$

$v_{jk}$ is designed as a function of $g_{jk}$ and $L_{jm},m=1,...,C$. In other terms, the proposed evaluation function takes into account the collective impact of all relative membership degrees $g_{jm},m=1,...,C$ corresponding to each 5QI $I_{j}$, by comparing them against the threshold and representing these degrees as logical values $L_{jm}$.

In the context of the three-branch decision partition, the selection of the threshold pair is associated with the definition of the variable $v_{jk}$. Specifically, when $I_{j}$ is an element of the CR of $af_{p}$, there exist $g_{jp}=1,L_{jp}=1,L_{jk}=0,k\neq p$. Thus, $v_{jp}=1$. When $I_{j}$ belongs to the BR of h classes (2≤h≤C), there exist $g_{jb}>\delta_{a},1>v_{jb}>\delta_{a}/h,b\in \left\{b_{1},...,b_{h}\right\}$. Therefore, the partitioning threshold pair $\left(\alpha_{j},\beta_{j}\right)$ is computed as follows:

(15) $$\alpha_{j}=1$$

(16) $$\beta_{j}=\frac{\delta }{\sum_{k=1}^{C}L_{jk}}$$

Finally, the region assignment is determined based on the evaluation function $v_{jk}$ between the element $I_{j}$ and the cluster center $c_{k}$, together with the threshold pair $\left(\alpha_{j},\beta_{j}\right)$. Specifically, if $v_{jk}\geq \alpha_{j}$, then $I_{j}$ is assigned to the core region $CR_{k}$; if $\alpha_{j}>v_{jk}>\beta_{j}$, then $I_{j}$ is assigned to the boundary region $BR_{k}$. Otherwise, $I_{j}$ is assigned to the negative region $DR_{k}$.

Compared to the distance-based partitioning method, the three-way decision-based approach takes into account the distribution of the relative membership degrees of each element with respect to all clustering centers. Moreover, it eliminates the need to manually configure the threshold pair parameters. Instead, the threshold $\beta_{j}$ for the boundary and negative regions is adaptively adjusted according to the distribution of relative membership degrees.

#### 3.2.2. Dynamic Weighted QoS Mapping in 5G-TSN Networks

**Figure 3:**
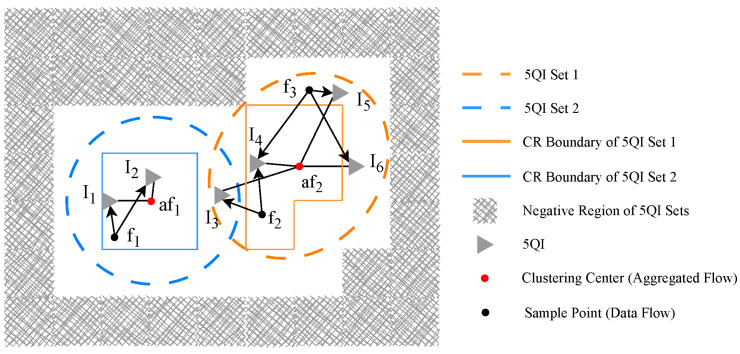
Mapping description based on clustering of TSN traffic flows and 5QI region partitioning.

After the TSN traffic clustering and 5QI region partitioning steps are completed, the final stage involves executing dynamic weighted QoS mapping. This mapping is performed from the TSN flows within each aggregated flow to the element of the 5QI region, following the sequence of “core region first, followed by boundary region.” In reference [53], a greedy approximate mapping strategy is adopted, which prioritizes the selection of the 5QI that exhibits the highest similarity to the aggregated traffic cluster center. As a result, the traffic load tends to concentrate on a limited number of 5QIs. Although greedy approximate mapping reduces computational complexity (C < N), this approach neglects the QoS differences between individual traffic flows and various 5QIs. Figure 3 shows a simplified illustration.

Two aggregated flows have some unmapped elements $\left\{f_{1},f_{2},f_{3}\right\}$. The two corresponding 5QI sets have some elements under different regions $<CR_{1}:\left\{I_{1},I_{2}\right\},BR_{1}:\left\{I_{3}\right\}>,<CR_{2}:\left\{I_{4}\right\},BR_{2}:\left\{I_{5},I_{6}\right\}>$.

When $CR_{1}$ (or $BR_{2}$) has remaining capacity, the greedy approximation mapping assigns $f_{1}$ (or $f_{3}$) to 5QI $I_{2}$ (or $I_{6}$). However, this results in significant QoS mismatches, as $f_{1}$ (or $f_{3}$) is actually closer to 5QI $I_{1}$ (or $I_{5}$). Furthermore, when $CR_{2}$ has remaining capacity, $f_{2}$ always prioritizes $I_{4}$ in the core domain.

In short, greedy approximate mapping may cause the issue of load concentration and QoS mismatch. To mitigate this potential issue, dynamic weighted QoS mapping is proposed. For each element $f_{ki}$ in the aggregated flow cluster, its dynamic weight with respect to a 5QI element $I_{j}$ in the core or boundary region is calculated as follows:

(17) $$w_{ki,j}^{c}=r\cdot \left(1-\frac{u_{i}}{N_{0}}\right)+\left(1-r\right)\cdot e^{-d_{ki,j}}$$

Here, *r* is the weighting factor, with a value range of 0≤r≤1; $N_{0}$ denotes the maximum allowable load for a single 5QI; $u_{j}$ represents the current load of the 5QI element $y_{j}$; and $d_{ki,j}$ is the normalized distance between $f_{ki}$ and $I_{j}$.

The first term of the dynamic weight $w_{ki,j}^{c}$ reflects the load residual ratio of $I_{j}$, while the second term captures the QoS similarity between $f_{ki}$ and $I_{j}$. To prevent any single factor from having an excessive impact on the mapping effect, an exponential function is used to calculate the similarity component in Equation (15), ensuring that the value ranges of both parts are approximately consistent within [0, 1]. These two components are combined through a weighted summation to derive the final dynamic weight. The mapping procedure proceeds as follows: first, the 5QI elements $I_{j}$ with non-zero residual capacity are selected; next, the dynamic weights for all eligible $I_{j}$ are computed; finally, $f_{ki}$ is mapped to the 5QI with the highest dynamic weight. This process repeats until all aggregated flows are mapped or until both the core and boundary regions have no remaining load capacity.

### 3.3. Time Complexity Analysis

In the TSN traffic clustering stage, the time complexity for computing the CRITIC–entropy weights is $O\left(N\right)$, while that for the Fisher criterion weights is $O\left(C\right)$. Therefore, the overall time complexity of the weighted FCM clustering is $O\left(N\cdot C+N+C\right)=O\left(N\cdot C\right)$. In the 5QI region partitioning stage, the time complexity of the three-way decision-based partitioning method is $O\left(4\cdot L\cdot C+4\cdot L+1\right)=O\left(L\cdot C\right)$ [54]. In the dynamic mapping stage, the total time complexity is $O\left(N\right)$. Consequently, the overall time complexity of the proposed WFCM-TDwQM algorithm is $\begin{array}{c} O(\max(N\cdot C,L\cdot C)) \end{array}$.

## 4. Simulation and Discussion of Results

### 4.1. Experimental Setup

In the 5G-TSN converged network, traffic load is defined as the number of service flows, which is set to a maximum of 630 in the simulation. Since a single PDU session can support up to 64 QoS flows, the 5G bridge is only required to establish a maximum of 10 PDU sessions. The load upper bound for a single 5QI is configured in relation to the number of PDU sessions.

In this study, the QoS requirements of the TSN traffic to be mapped are generated based on nine types of industrial network traffic data provided in Table 2 of [31,33]. The 5QI set used for mapping includes both standard 5QIs defined in 3GPP Release [41] and extended non-standard 5QIs derived from the mapping results. The validation is conducted from two perspectives: (1) under the same network scenario, varying traffic loads are evaluated, ranging from 150 to 630 flows; (2) under a fixed traffic load, different network scenarios are considered, as shown in Table 2, where the classification of TSN traffic types follows Table 1. The simulation experiments in this study evaluate four algorithm combinations: WFCM-TDwQM, IKM-RQM, WFCM-RQM, and IKM-TDwQM. These combinations are derived by integrating the proposed WFCM-TDwQM algorithm with the IKM-RQM method [31]. The key parameters used in the simulations are listed in Table 3. The performance evaluation metrics include the mapping success rate, the proportion of flows meeting the packet loss rate requirement, the mapping error, the load coverage ratio, the average length of non-empty loads, and the load variance (only non-empty load).

### 4.2. Determination of Cluster Number and Weighting Parameter

#### 4.2.1. Cluster Count C

**Figure 4:**
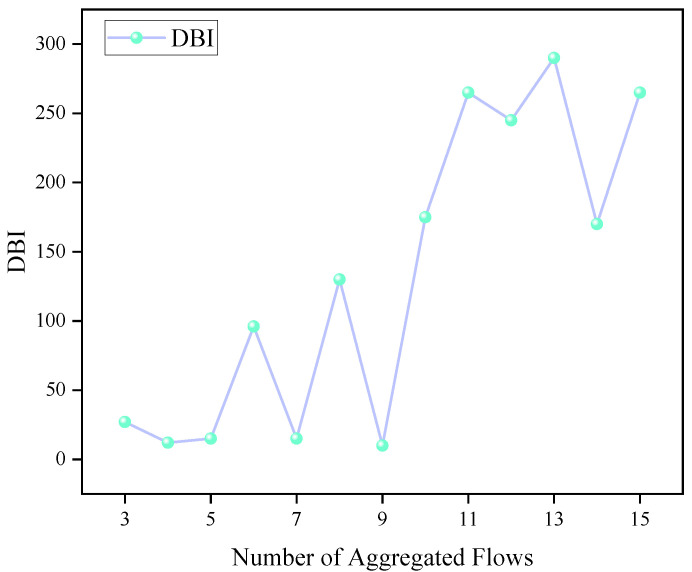
DBI values under different numbers of aggregated flows.

A reasonable number of clustering centers (i.e., aggregated flows) is essential for improving clustering performance. In this study, the Davies–Bouldin Index (DBI) is employed to evaluate clustering quality under different numbers of cluster centers, where a smaller DBI value indicates better clustering performance. As shown in Figure 4, the DBI value increases as the number of aggregated flows increases. The DBI reflects the average of the maximum ratios between intra-cluster distance and inter-cluster distance. If the number of aggregated flows is too small, each cluster will contain too many traffic flows, which may cause network congestion. On the other hand, too many clusters will increase computational overhead. Therefore, the number of aggregated flows is set to 9 in this study, which corresponds to a relatively low DBI value.

#### 4.2.2. Proportion Parameter r

**Figure 5:**
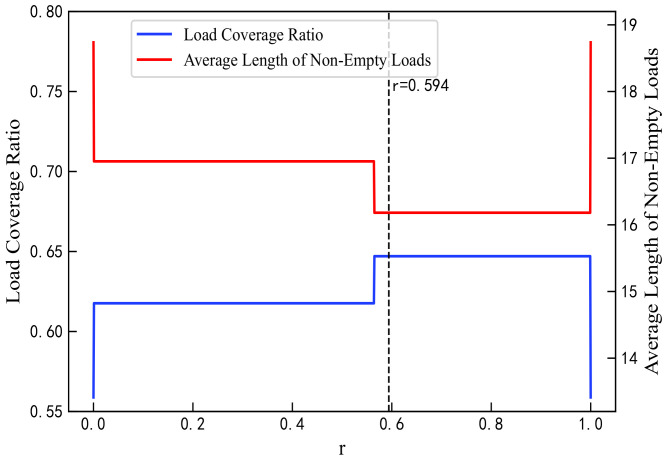
Load coverage ratio and average length of non-empty loads under different values of r.

**Figure 6:**
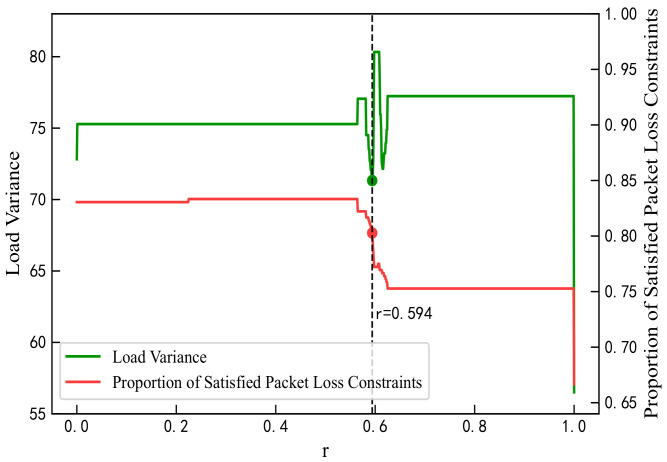
Load variance and packet loss satisfaction ratio under different values of r.

**Figure 7:**
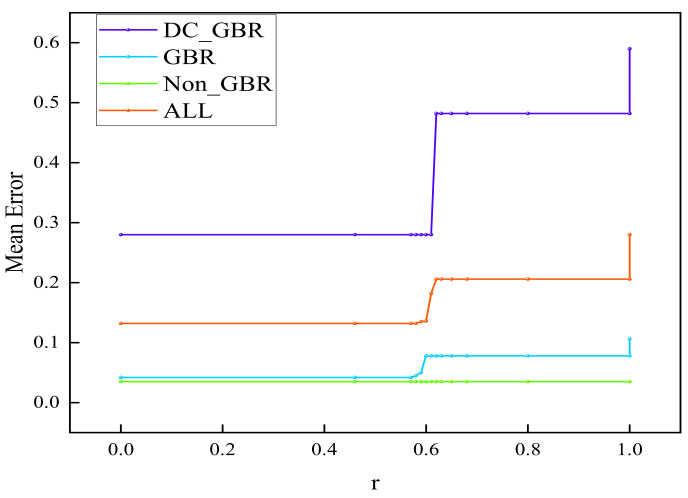
Average mapping error of DC-GBR, GBR, non-GBR, and overall under different values of r.

The proportion parameter *r* for QoS mapping must be predetermined. In this study, an optimal value of *r* was selected by analyzing the mapping results of the proposed algorithm when N=360. As *r* increases from 0 to 1 in Figure 5, the residual load weight rises, leading to improvements in load coverage and reductions in average load length, thus enhancing load balance and effectively mitigating network congestion. As shown in Figure 6, the overall load variance decreases, reaching its minimum at r=0.594 except at the boundary values. Packet loss rates decline within the range r=0.58 to 0.625, with the rate at r=0.594 maintaining at least 80%. As shown in Figure 7, errors in the GBR/DC-GBR categories increase due to the increase in load balancing weight, whereas the non-GBR errors remain stable, all at relatively low levels when r=0.594. Taking into account both load balance and error control, the value r=0.594 was ultimately chosen. Furthermore, the mapping success rate remains constant, attributed to the fixed clustering and regional partitioning results, which effectively constrain the impact of variations in *r*.

**Figure 8:**
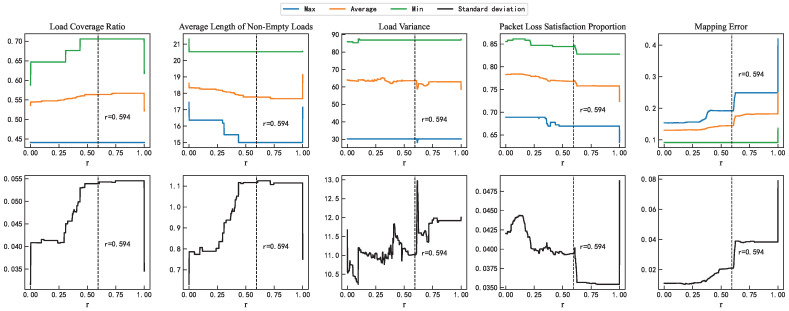
Comparison of mapping performance with different values of r.

To verify the robustness of the empirically determined value r = 0.594 in terms of performance, this study repeated each possible value of r 100 times. At r = 0.594, in Figure 8, the load coverage ratio and the average length of non-empty loads are located well within the stable range (particularly near the middle of the average values), with the remainder metric positioned at the left end of its stable range, indicating good performance stability. Regarding the variability in performance metrics at r = 0.594, the first two metrics exhibit larger standard deviations—approximately 5.5% and 1.1, respectively—due to the effects of load balancing; the other metrics maintain moderate standard deviations of 11%, 4%, and 0.21, respectively.

### 4.3. Comparison of Multiple Algorithms Under Varying Loads in Single Network Scenario

Under the traffic distribution of the general Scenario 2, the network load is gradually increased to evaluate the mapping performance of four different algorithm combinations. The network load ranges from 150 to 630, with an increment of 30. The comparison focuses on two aspects: QoS consistency and load balancing. For QoS consistency, the evaluation metrics include the mapping success rate, the proportion of flows meeting the packet loss rate requirement, and the mapping error. For load balancing, the metrics include the load coverage ratio, the average length of non-empty loads, and the load variance.

**Figure 9:**
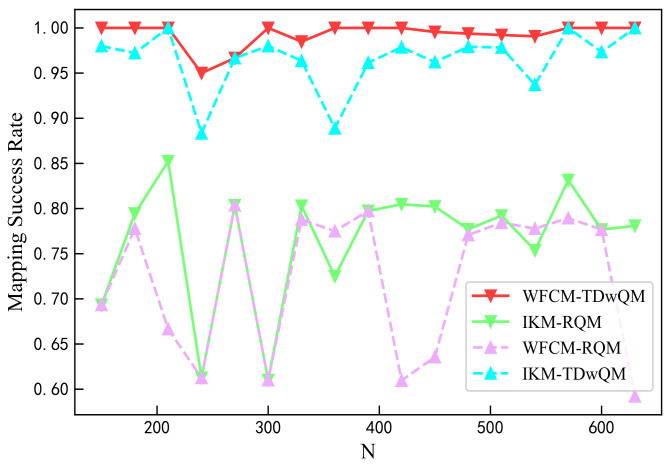
Comparison of mapping success rate.

As the network load increases, the QoS consistency metrics of the four algorithm combinations are examined. As shown in Figure 9, when the network load increases from 150 to 630, the proposed WFCM-TDwQM algorithm achieves the highest mapping success rate, with an average of 99.3%. In comparison, IKM-TDwQM ranks second with an average of 96.5%, while IKM-RQM and WFCM-RQM perform worse, with average success rates of 76.5% and 72.1%, respectively.

**Figure 10:**
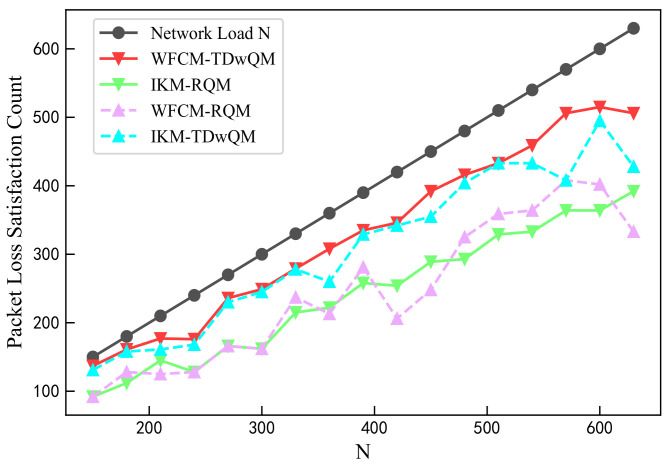
Comparison of packet loss satisfaction count.

As illustrated in Figure 10, among all four algorithm combinations, WFCM-TDwQM consistently achieves the highest number of flows satisfying the packet loss rate requirement—especially under medium- to high-load conditions—demonstrating its superior performance in both success rate and reliability.

**Figure 11:**
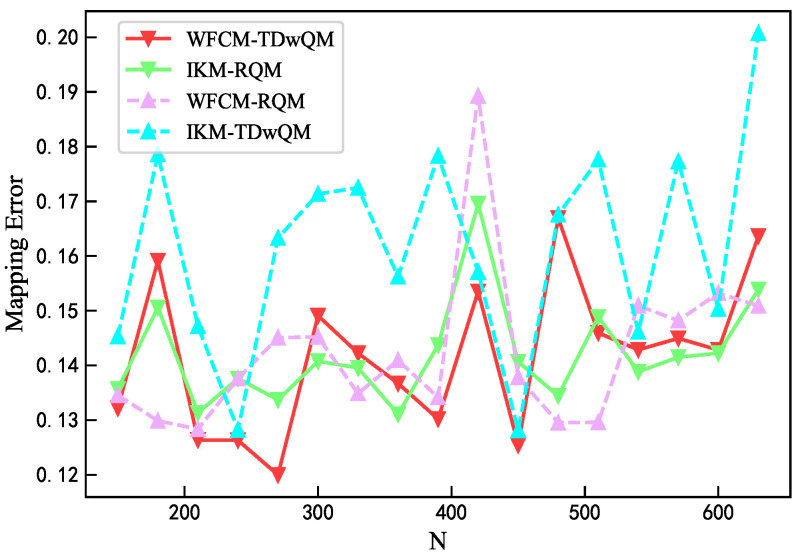
Comparison of mapping error.

According to Figure 11, the maximum mapping error of WFCM-TDwQM does not exceed 0.164, and its overall error variation is better than that of IKM-TDwQM. In most cases, the remaining two algorithms exhibit lower individual mapping errors, but their performance is less stable overall.

**Figure 12:**
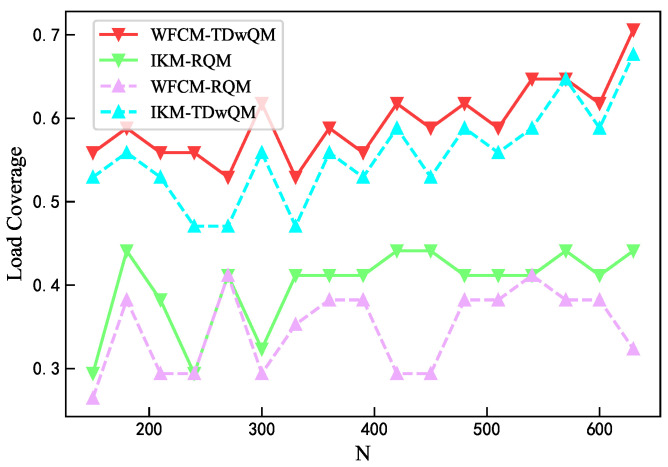
Comparison of load coverage ratio.

**Figure 13:**
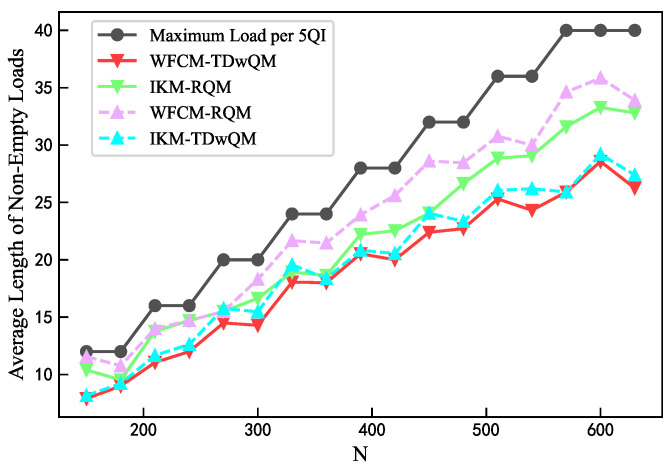
Comparison of average length of non-empty loads.

**Figure 14:**
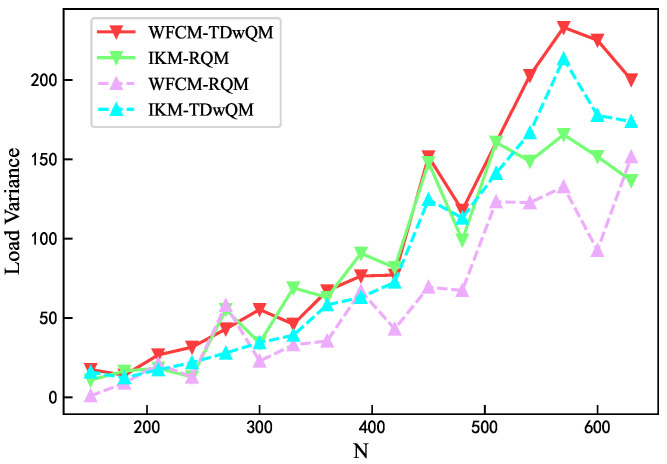
Comparison of load variance.

As the network load increases, the load balancing metrics of the four algorithm combinations are analyzed. As shown in Figure 12, WFCM-TDwQM consistently achieves the highest 5QI load coverage ratio, with an average of 59.5%, followed by IKM-TDwQM at 55.5%. The other two methods, IKM-RQM and WFCM-RQM, show significantly lower averages of 40.0% and 34.8%, respectively. As illustrated in Figure 13, the average non-empty load per 5QI increases with the rising network load. Overall, the values follow the order WFCM-RQM > IKM-RQM > IKM-TDwQM > WFCM-TDwQM, indicating that WFCM-TDwQM distributes the load more evenly across multiple 5QIs. According to Figure 14, the load variance across the 5QI set also shows an upward trend as the load increases. In most cases, WFCM-TDwQM exhibits a higher load variance. This is because a greater number of 5QIs are involved in the load balancing process, resulting in a wider but more effective distribution of traffic load.

**Figure 15:**
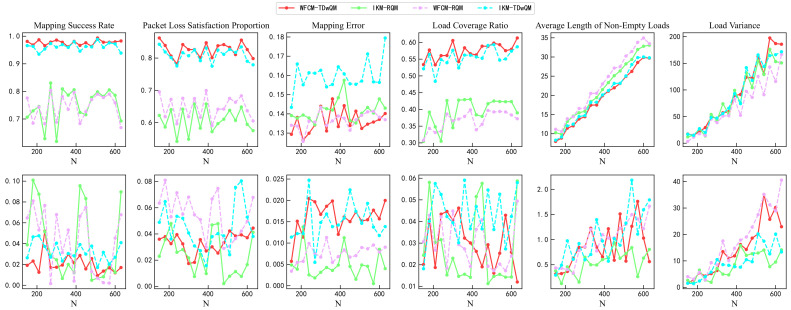
Comparison of mapping performance from multiple simulation runs.

For verification from the perspective of statistical significance, this study repeated each algorithm 100 times under various load conditions. As shown in Figure 15, the simulation results include the mean and standard deviation of multiple runs for all key performance indicators. In the first row of Figure 15, the changes in the mean values of the indicators further validate the conclusions presented earlier in Section 4.3. The second row of Figure 15 shows the changes in the standard deviations of the indicators. WFCM-TDwQM demonstrates better stability in the mapping success rate and packet loss satisfaction metrics, while the remaining indicators exhibit some fluctuations as the load increases.

Compared to the other three algorithm combinations, WFCM-TDwQM demonstrates a higher success rate in QoS mapping and a greater number of instances meeting packet loss rate requirements, as well as accommodating a larger volume of 5QI traffic. While maintaining QoS consistency, the resulting mapping scheme yields a lower average load per individual 5QI, indicating that the proposed algorithm exhibits enhanced adaptability to variations in network load.

### 4.4. Multi-Scenario Comparison of Multiple Algorithms Under Varying Load Levels

Different applications in the network impose varying requirements on traffic types, resulting in changes in traffic composition across different scenarios, as shown in Table 2. For example, in Scenario 1, which has strict real-time performance requirements, a higher proportion of Type A traffic is observed. In contrast, Scenario 3, which represents audio–video services with high bandwidth demand, exhibits a larger proportion of Type B traffic.

Under the same network load condition (N=600), the mapping performance of the four algorithm combinations is compared across different network scenarios. The performance metrics are consistent with those used in Experiment 2. In contrast to the perspective of Experiment 2, which emphasizes variations in network load, Experiment 3 investigates the impact of different traffic compositions across network scenarios on algorithm performance.

**Figure 16:**
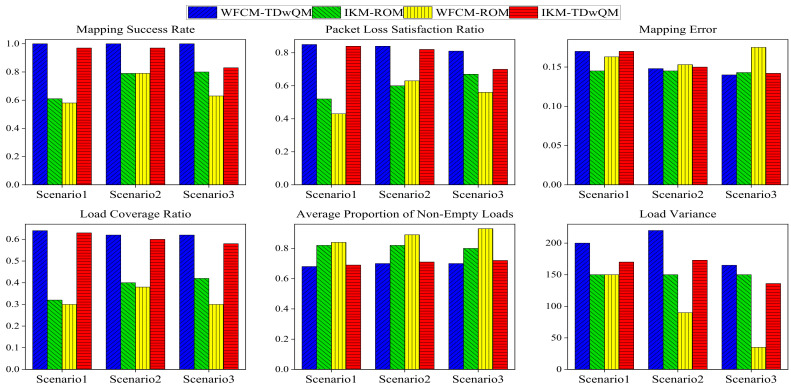
Comparison of mapping performance of four algorithm combinations across multiple scenarios under same network load N = 600.

As shown in Figure 16, in the audio–video-oriented Scenario 3, the proposed WFCM-TDwQM algorithm not only ensures QoS consistency between pre- and post-mapping—reflected in its high mapping success rate, high proportion of flows meeting the packet loss constraint, and low mapping error—but also achieves excellent load distribution performance, including high load coverage, a low average proportion of non-empty loads, and a reasonable level of load variance.

**Figure 17:**
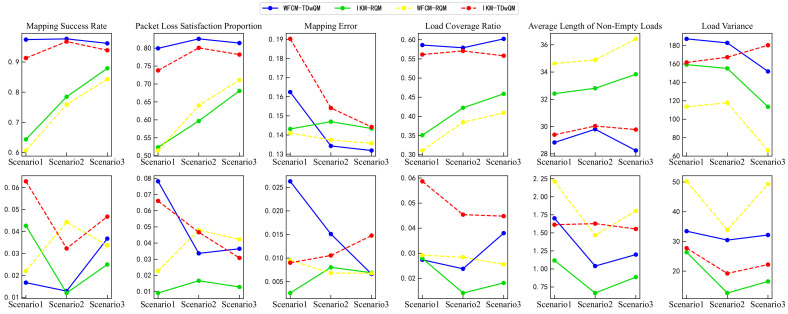
Comparison of mapping performance from multiple simulation runs across multiple scenarios.

The experiment was also repeated 100 times. In the first row of Figure 17, the changes in the mean values of the metrics further validate the conclusions presented earlier in Section 4.4. The second row of Figure 17 shows the changes in the standard deviations of the metrics. As the proportion of non-time-sensitive traffic increases, WFCM-TDwQM exhibits an increasing trend in performance variability for the metrics mapping success rate and load coverage ratio; it shows relatively stable fluctuations for load variance; and a decreasing trend in variability for the remaining metrics.

Compared across the four algorithm combinations and three different scenarios, the proposed algorithm consistently achieves a mapping success rate close to 100%, a packet loss satisfaction rate no lower than 80%, a load coverage ratio of at least 60%, and an average non-empty load ratio not exceeding 75%. Therefore, the proposed algorithm demonstrates both high QoS mapping fidelity and favorable load distribution across network scenarios with diverse traffic requirements, maintaining stable performance even as traffic composition varies.

## 5. Conclusions

This paper addresses the QoS mapping problem in 5G-TSN converged networks, with a focus on ensuring both QoS consistency and balanced load distribution. A dynamic weighted QoS mapping algorithm, termed WFCM-TDwQM, is proposed by integrating weighted fuzzy C-means (WFCM) clustering and three-way decision theory. The algorithm clusters TSN traffic based on QoS attributes using WFCM, then employs a three-way decision-based partitioning strategy and dynamic weight mapping to adaptively adjust the 5QI set and mapping weights in response to changing network conditions, thereby improving the performance of the QoS mapping scheme. The simulation results demonstrate that WFCM-TDwQM outperforms baseline algorithms in key metrics such as mapping success rate, the proportion of flows satisfying packet loss constraints, load coverage, and the average length of non-empty loads—especially under varying network loads and traffic compositions. These findings confirm that the proposed method not only maintains QoS consistency in the mapping process but also achieves effective load balancing.

It is worth noting that the clustering stage in the proposed method currently relies on predefined optimal cluster numbers, which are obtained through prior data analysis. In future work, we aim to explore more scalable and adaptive traffic clustering algorithms that can better accommodate dynamic network environments and a broader range of traffic types.

## Figures and Tables

**Table 1:** QoS class mapping and metric mapping.

TSN Traffic Types	PCP of TSN Traffic	5QI Resource Type
Class A (high priority)	7, 6, 5, 4, 3	DC-GBR
Class B (medium priority)	2, 1	GBR
Class C (low priority)	0	NON-GBR

**Table 2:** Simulation scenario configuration.

	TSN Traffic Proportion (%)	TSN Traffic Type Distribution (%)
Scenario 1	3:7:7:6:3:5:9:9:51	26:23:51
Scenario 2	2:6:6:5:2:8:15:15:41	21:38:41
Scenario 3	2:6:6:5:2:10:19:19:31	21:48:31

**Table 3:** Simulation parameters.

Parameter	Value
Maximum Number of PDU Sessions	10
Load Upper Bound per 5QI	4× Number of PDU Sessions
Clustering Convergence Threshold	0.01
Minimum Distance Threshold	0.05
Relative Distance Threshold	1.5

## Data Availability

For access to the data used in this study, please contact the corresponding author if needed.
